# Breast Cancer Cell-Derived Exosomal miR-92b-3p Promotes Tumor Angiogenesis and Metastasis by Suppressing PTEN in Vascular Endothelial Cells

**DOI:** 10.32604/or.2026.083563

**Published:** 2026-07-16

**Authors:** Tingting Yang, Meng Guan, Xin Guan, Lihua Kang, Xiaomeng Wang, Yanjie Guan, Yang Yang, Wei Deng, Guoxiang Wang

**Affiliations:** 1 Cancer Center, First Hospital of Jilin University, Jilin University, Changchun, China; 2 Department of Hematology and Oncology, China-Japan Union Hospital of Jilin University, Jilin University, Changchun, China; 3 Engineering Research Center for Digital Medicine and Health of Guangxi Higher Education Institutions, Guangxi Medical University, Nanning, China

**Keywords:** Breast cancer, exosomes, miR-92b-3p, angiogenesis, vascular permeability

## Abstract

**Background:** Tumor-driven vascular remodeling is crucial for breast cancer metastasis; yet, the role of tumor-derived exosomal miRNAs in this process remains underexplored. This study aimed to investigate the clinical relevance and the underlying mechanism of breast cancer-derived exosomal miR-92b-3p in endothelial reprogramming. **Methods:** miR-92b-3p expression was evaluated in the TCGA cohort and clinical patient samples. The effects of exosomal miR-92b-3p from breast cancer cells on recipient human microvascular endothelial cells (HMVECs) were assessed using *in vitro* angiogenesis, migration, and permeability assays, alongside *in vivo* murine xenograft models. Mechanistic targets were validated via dual-luciferase and rescue experiments. **Results:** miR-92b-3p was significantly upregulated in breast cancer tissues and plasma exosomes, correlating with advanced stages, poor survival, and exhibiting high diagnostic accuracy. Breast cancer-derived exosomes effectively transferred miR-92b-3p into HMVECs, significantly promoting angiogenesis, endothelial migration, and transendothelial permeability. *In vivo*, overexpression of miR-92b-3p accelerated tumor growth, vascularization, and circulating tumor cell (CTC) dissemination. Mechanistically, exosomal miR-92b-3p directly targeted and suppressed PTEN in endothelial cells; moreover, restoring PTEN expression fully abrogated the exosome-induced pro-angiogenic and hyperpermeable phenotypes. **Conclusions:** Breast cancer-derived exosomal miR-92b-3p disrupts the vascular barrier and promotes tumor angiogenesis by targeting endothelial PTEN, thereby facilitating metastasis. Circulating exosomal miR-92b-3p represents a promising candidate liquid-biopsy biomarker for breast cancer progression.

## 1 Introduction

Breast cancer remains the most frequently diagnosed malignancy in women worldwide and a leading cause of cancer-related death [1]. Although substantial advances have been made in surgery, endocrine therapy, chemotherapy, radiotherapy, and targeted treatment, metastatic dissemination continues to be the major determinant of poor clinical outcome [2,3]. Angiogenesis is central to this process: newly formed vessels not only support tumor growth but also provide the anatomical and functional basis for tumor cell intravasation and systemic spread. Importantly, the vascular contribution to metastasis is not limited to increased vessel number. Endothelial remodeling and barrier dysfunction are now recognized as critical features of the metastatic microenvironment. Given the marked molecular heterogeneity of breast cancer, mechanisms that influence vascular behavior across distinct subtype backgrounds are of particular interest [4,5,6].

Exosomes are 30–150 nm extracellular vesicles secreted by most cell types that contain proteins, lipids, and nucleic acids and mediate intercellular communication [7,8,9]. In cancer, exosomal microRNAs (miRNAs) have emerged as important regulators of immune modulation, therapeutic resistance, angiogenesis, and metastasis through the transfer of regulatory signals from tumor cells to recipient cells within the tumor microenvironment [10,11,12]. Among these molecules, miR-92b-3p has been reported to be aberrantly expressed in several malignancies, including pancreatic cancer, esophageal squamous cell carcinoma, colorectal cancer, and gastric cancer, although its functional role appears to be context dependent [13,14,15,16]. In breast cancer, elevated circulating miR-92b-3p has been associated with unfavorable clinicopathological features [17]. However, whether breast cancer cells package miR-92b-3p into exosomes and use it to reprogram vascular endothelial cells has not been clearly established.

PTEN is a key negative regulator of PI3K/AKT signaling and plays broad roles in cell survival, migration, angiogenesis, and junctional stability [18,19,20]. Previous studies have shown that miR-92b-3p can directly target PTEN in glioma, thereby enhancing AKT signaling and promoting malignant phenotypes. These observations raise the possibility that, in breast cancer, miR-92b-3p may act not only through tumor cell-intrinsic pathways but also through exosome-mediated remodeling of the vascular microenvironment. Such a mechanism would be especially relevant to endothelial permeability, because PTEN loss has been linked to pro-angiogenic signaling and impaired endothelial junction integrity. From this perspective, exosomal miR-92b-3p may represent not merely a differentially expressed circulating miRNA, but a functionally relevant mediator of endothelial activation and tumor dissemination.

On this basis, the present study investigated breast cancer-derived exosomal miR-92b-3p from clinical, functional, and mechanistic perspectives. We first examined its expression in the TCGA-BRCA cohort and in patient plasma, plasma exosomes, and tissue samples, with attention to its association with disease stage and its potential value as a liquid-biopsy candidate. We then evaluated whether tumor-derived exosomal miR-92b-3p can be transferred to vascular endothelial cells and whether this transfer affects endothelial migration, angiogenesis, and permeability-related transendothelial tumor cell migration. Finally, we assessed whether PTEN serves as a key functional mediator of these effects. By focusing on exosomal delivery to endothelial cells, this study seeks to define a more specific mechanism by which breast cancer reshapes the vascular microenvironment during progression, while recognizing that the downstream signaling network and the clinical utility of this marker will require further validation.

## 2 Materials and Methods

### 2.1 Tumor Tissue and Plasma Samples

Plasma samples were collected from 60 patients with breast cancer, 10 patients with benign breast disease, and 10 healthy controls at the First Hospital of Jilin University. The breast cancer cohort included 15 cases each of clinical stage I, II, III, and IV disease. In addition, paired cancerous and paracancerous tissues were obtained from 10 breast cancer patients who underwent surgery at The First Hospital of Jilin University between January and December 2022. This study was based on a single-center cohort, and an independent validation cohort was not available. Available clinicopathological information was retrieved from the medical records where possible. The available clinical characteristics of the participants included in the plasma and plasma exosome cohort are summarized in Table 1. All samples were aliquoted and stored at −80°C until analysis. The study was conducted in accordance with the Declaration of Helsinki of 1975 (as revised in 2013), and the protocol was reviewed and approved by the Ethics Committee of the First Hospital of Jilin University (2023-KS-355), with written informed consent obtained from all participants before enrollment.

**Table 1:** Clinical characteristics of participants included in the plasma and plasma exosome cohort.

Variable	Breast Cancer Patients (n = 60)	Benign Breast Disease Patients (n = 10)	Healthy Controls (n = 10)
Age, years, median (range)	50 (35–65)	56 (45–65)	48 (35–65)
Clinical stage (AJCC), n (%)		—	—
Stage I	15 (25.0)	—	—
Stage II	15 (25.0)	—	—
Stage III	15 (25.0)	—	—
Stage IV	15 (25.0)	—	—
ER status, n (%)		—	—
Positive	38 (63.3)	—	—
Negative	22 (36.7)	—	—
PR status, n (%)		—	—
Positive	28 (46.7)	—	—
Negative	32 (53.3)	—	—
HER2 status, n (%)		—	—
Positive	26 (43.3)	—	—
Negative	34 (56.7)	—	—
Blood samples collected before treatment, n (%)		—	—
Yes	60 (100.0)	—	—
No	0 (0.0)	—	—
Unknown	—	—	—
Benign diagnosis, n (%)	—	10 (100.0)	—
Fibroadenoma	—	9 (90.0)	—
Other benign breast lesions	—	1 (10.0)	—

Data are presented as n (%) unless otherwise indicated. Blank cells indicate data not available; “—” indicates not applicable.

### 2.2 Cell Culture

The cell lines used in this study included human embryonic kidney cells 293T, human mammary epithelial cells MCF-10A, human microvascular endothelial cells HMVECs, human breast cancer cell lines MCF7 (National Collection of Authenticated Cell Cultures, SCSP-531, Shanghai, China), T-47D (National Collection of Authenticated Cell Cultures, SCSP-564, Shanghai, China), ZR-75-1 (National Collection of Authenticated Cell Cultures, TCHu126, Shanghai, China), MDA-MB-231 (National Collection of Authenticated Cell Cultures, SCSP-5043, Shanghai, China), SK-BR-3 (National Collection of Authenticated Cell Cultures, SCSP-5243, Shanghai, China), MDA-MB-453 (National Collection of Authenticated Cell Cultures, SCSP-5044, Shanghai, China), and HCC1954 (National Collection of Authenticated Cell Cultures, TCHu245, Shanghai, China), and the mouse endothelial cell line C166 (National Collection of Authenticated Cell Cultures, SCSP-5488, Shanghai, China). 293T cells (BMCR, 3101HUMGNHu17, Beijing, China) were obtained from the National Biomedical Experimental Cell Resource Bank/Basic Medical Cell Resource Center, Chinese Academy of Medical Sciences & Peking Union Medical College. MCF-10A, HMVECs, MCF7, T-47D, ZR-75-1, MDA-MB-231, SK-BR-3, MDA-MB-453, C166 and HCC1954 cells were obtained from the Cell Bank of the Chinese Academy of Sciences (NCACC; Shanghai, China).

All cell lines were cultured according to the recommendations provided by the corresponding cell banks. Human cell lines were authenticated by short tandem repeat (STR) profiling by the suppliers before use, and the identity of the cell lines was confirmed to be consistent with the corresponding reference profiles. For HMVECs and C166 cells, cell identity and quality control information were verified according to the certificates provided by the cell resource centers. All cells were routinely tested for mycoplasma contamination using a PCR-based mycoplasma detection assay before experiments and during routine culture, and all cells used in this study were negative for mycoplasma contamination.

MCF7 and MDA-MB-231 cell lines with different expression levels of miR-92b-3p were generated using Lipofectamine 3000 (Thermo Fisher Scientific, L3000008, Waltham, MA, USA). Fluorescence microscopy (Leica DMil; Leica Microsystems, 11526310, Wetzlar, Germany) was used to visualize fluorescence signals, and stable transgenic cell lines were selected with G418 antibiotic (500 μg/mL) (Thermo Fisher Scientific, 10131035, Waltham, MA, USA) for four weeks. Before exosome-related experiments, exosomes were depleted from fetal bovine serum by ultracentrifugation to prepare exosome-free FBS (Gibco/Thermo Fisher Scientific, 10099141C) and to avoid interference from serum-derived extracellular vesicles.

### 2.3 Analysis of the BRCA Cohort Using the Cancer Genome Atlas (TCGA) Database

Breast cancer data were obtained from the TCGA-BRCA project in the National Cancer Institute Genomic Data Commons (GDC) Data Portal (https://portal.gdc.cancer.gov/projects/TCGA-BRCA). miRNA expression quantification files and corresponding clinical metadata were downloaded from the GDC portal. Only primary breast invasive carcinoma samples and solid tissue normal samples with available miRNA-seq data were included in the differential expression analysis. Samples lacking miRNA expression data, pathological stage information, or survival information were excluded from the stage-based and survival analyses. When multiple aliquots were available from the same patient, only one representative sample was retained to avoid duplication. Raw count data were processed in R (version 3.3.3) using stringr (v. 1.2.0), tidyverse (v. 1.1.1), miRBaseVersions.db (v21), edgeR (v. 3.16.5), and ggplot2 (v. 2.2.1). Low-abundance miRNAs were filtered by retaining only those with counts per million (CPM) > 1 in at least 20% of the samples; miRNAs not meeting this criterion were removed before downstream analysis. Expression counts were normalized using the edgeR package. Differential expression between tumor and normal breast tissues was analyzed in edgeR, and *p* values were adjusted using the Benjamini-Hochberg method; miRNAs with a false discovery rate (FDR) < 0.05 and Log2 |FC| > 1 were considered differentially expressed. For clinical stage analysis, pathological stages were harmonized into stages I, II, III, and IV according to the TCGA annotations. To evaluate the relationship between miR-92b-3p and disease progression, two complementary analyses were performed: an overall comparison across stages I–IV and an exploratory grouped comparison between stages I–III and stage IV, with stage IV representing overt metastatic disease. Volcano plots and other data visualizations were generated using ggplot2.

No additional clinical exclusion criteria were applied because TCGA is a publicly available de-identified database.

### 2.4 Isolation and Identification of Exosomes

For tissue-derived extracellular vesicles, fresh tissue fragments were digested with type I collagenase (1 mg/mL; Sigma, USA) at 37°C for 45 min. The digestion was stopped on ice, protease/phosphatase inhibitors (Yeasen Biotechnology, 20124ES03, Shanghai, China) were added, and the supernatant was subjected to sequential centrifugation at 4°C to remove cells and debris (3000× *g* for 20 min, followed by 10,000× *g* for 30 min). The clarified supernatant was passed through a 0.22 μm filter and ultracentrifuged at 110,000× *g* for 70 min. The pellet was washed once with PBS and centrifuged again under the same conditions before final resuspension in PBS. Plasma-derived vesicles were isolated using a similar differential ultracentrifugation protocol. Briefly, plasma was first centrifuged at 3000× *g* for 10 min and 10,000× *g* for 30 min to remove cells, debris, and large vesicles. The supernatant was then filtered through a 0.22 μm membrane, ultracentrifuged at 110,000× *g* for 70 min, washed once with PBS, and centrifuged again before resuspension in PBS. Throughout the manuscript, these ultracentrifugation-based preparations are referred to as exosome-enriched vesicles.

Exosome-enriched preparations were characterized by transmission electron microscopy (TEM), nanoparticle tracking analysis (NTA), and Western blotting before use in downstream assays. TEM was performed using a Tecnai G2 SPIRIT microscope (FEI Company, Tecnai G2 SPIRIT, Hillsboro, OR, USA) to assess vesicle morphology, and NTA was performed on a NanoSight NS300 system (Malvern Panalytical, NTA300, Malvern, UK) to determine particle size distribution and concentration. Western blotting was used to detect the canonical vesicle-associated proteins CD9 (CD9 antibody, Abcam, ab236630, Cambridge, UK), CD63 (CD63 antibody, Abcam, ab134045, Cambridge, UK), and TSG101 (TSG101 antibody, Abcam, ab125011, Cambridge, UK), together with the negative markers Calnexin (Calnexin antibody, Abcam, ab22595, Cambridge, UK) and Cytochrome C (Cytochrome C antibody, Abcam, ab133504, Cambridge, UK) (β-actin antibody, Sigma-Aldrich, A5441, St. Louis, MO, USA). Preparations showing a predominant particle distribution within the expected size range and the expected marker profile were used for subsequent experiments. In functional assays, vesicle input was normalized primarily by particle number as determined by NTA.

### 2.5 RT-qPCR

Total RNA from cells and tissues was extracted using TRIzol™ Reagent (Invitrogen/Thermo Fisher Scientific, 15596026, Carlsbad, CA, USA), and miRNA from plasma and exosome-enriched vesicles was extracted using the miRNeasy Serum/Plasma Kit (QIAGEN, 217184, Hilden, Germany) or miRNeasy Micro Kit (QIAGEN, 217084, Hilden, Germany) with QIAzol Lysis Reagent (QIAGEN, 79306, Hilden, Germany), according to the manufacturers’ instructions. RNA concentration and purity were measured using a NanoReady micro-volume analyzer (Agilent Technologies, Agilent 5300, Santa Clara, CA, USA). 

Samples with A260/A280 ratios of 1.8–2.1 were considered acceptable for subsequent reverse transcription. RNA integrity was assessed for cell- and tissue-derived total RNA by agarose gel electrophoresis and, when sample amount permitted, using an Agilent 2100 Bioanalyzer (Agilent Technologies, G2939A, Santa Clara, CA, USA); plasma RNA and exosome-derived RNA were not subjected to integrity assessment because of their low abundance and small-RNA-enriched profile. 

For each reverse transcription reaction, 1 μg of RNA was used. Genomic DNA was first removed in a 10 μL reaction containing 2.0 μL 5× gDNA Eraser Buffer, 1.0 μL gDNA Eraser, 1 μg RNA, and RNase-free dH_2_O to 10 μL, using PrimeScript™ RT reagent Kit with gDNA Eraser (Takara Bio, RR047A, Kusatsu, Shiga, Japan). The genomic DNA removal reaction was performed at 42°C for 2 min and then held at 4°C. Reverse transcription was then performed in a 20 μL reaction containing 10 μL of the genomic DNA removal reaction mixture, 1.0 μL PrimeScript RT Enzyme Mix I, 1.0 μL RT Primer Mix or miRNA-specific stem-loop RT primer, 4.0 μL 5× PrimeScript Buffer 2, and 4.0 μL RNase-free dH_2_O. The reverse transcription conditions were 37°C for 15 min, 85°C for 5 s, and 4°C hold. RT-qPCR was performed using TB Green^®^ Premix Ex Taq™ II (Tli RNaseH Plus) (Takara Bio, RR820A, Kusatsu, Shiga, Japan) on an ABI 7500 Real-Time PCR System (Applied Biosystems/Thermo Fisher Scientific, 7500, Foster City, CA, USA). Each 10 μL PCR reaction contained 5.0 μL 2× TB Green Premix Ex Taq II, 0.4 μL forward primer, 0.4 μL reverse primer, 0.2 μL ROX Reference Dye II, 2.0 μL cDNA template, and 2.0 μL sterile water. The cycling program was as follows: 95°C for 30 s, followed by 40 cycles of 95°C for 5 s and 60°C for 34 s. U6 was used as the internal reference for miRNA detection, and β-actin was used as the internal reference for mRNA detection. All primers were synthesized by Sangon Biotech (custom oligonucleotides, Shanghai, China). The primer sequences were as follows: U6 forward, 5′-CTCGCTTCGGCAGCACA-3′ and reverse, 5′-AACGCTTCACGAATTTGCGT-3′; miR-92b-3p stem-loop RT primer, 5′-GTCGTATCCAGTGCAGGGTCCGAGGTATTCGCACTGGATACGACGGAGGC-3′, forward, 5′-GCGTATTGCACTCGTCCCG-3′ and reverse, 5′-AGTGCAGGGTCCGAGGTAT-3′; β-actin forward, 5′-TTGCCCTGAGGCTCTTT-3′ and reverse, 5′-CATACAGGTCTTTGCGGATG-3′; PTEN forward, 5′-TTTGAAGACCATAACCCACCAC-3′ and reverse, 5′-ATTACACCAGTTCGTCCCTTTC-3′; and VE-cadherin forward, 5′-GGTCGATGCAGAGACAGGAG-3′ and reverse, 5′-GAGTCTCCAGGTTTTCGCCA-3′. Relative gene expression was calculated using the 2^−ΔΔCt^ method.

### 2.6 Exosome Uptake Assay

To evaluate exosome uptake, HMVECs were seeded in 24-well plates at a density of 5 × 10^4^ cells per well and cultured overnight in complete medium at 37°C in a humidified incubator with 5% CO_2_. Then, 200 μL of exosome suspension (2 × 10^11^ particles/mL) was labeled with 15 μM Vybrant™ DiO Cell-Labeling Solution (Thermo Fisher Scientific, V22886, Waltham, MA, USA) for 30 min at room temperature in the dark with gentle mixing, followed by ultracentrifugation at 110,000× *g* for 70 min at 4°C. The pellet was resuspended in PBS and added to the HMVECs for 24 h of co-culture. Cells treated with PBS alone and cells exposed to DiO-only preparations processed in parallel served as negative controls. After co-culture, the cells were washed three times with PBS for 5 min each to remove noninternalized exosomes, fixed with 4% paraformaldehyde fixation solution (Beyotime Biotechnology, P0099, Shanghai, China) for 15 min at room temperature, and washed three additional times with PBS. The nuclei were then counterstained with DAPI (Beyotime Biotechnology, C1002, Shanghai, China) at 1 μg/mL for 10 min in the dark. After a final PBS wash, fluorescence images were acquired using a Leica DMi1 fluorescence microscope (Leica Microsystems, 11526310).

### 2.7 GW4869 Treatment

To inhibit exosome biogenesis and release, MCF7 and MDA-MB-231 breast cancer cells were incubated with GW4869 (10 μM; Sigma-Aldrich, D1692, St. Louis, MO, USA), which was dissolved in dimethyl sulfoxide (DMSO; Sigma-Aldrich, D2650, St. Louis, MO, USA), for 24 h in complete medium consisting of high-glucose Dulbecco’s Modified Eagle’s Medium (DMEM; Gibco/Thermo Fisher Scientific, C11995500BT, Grand Island, NY, USA) supplemented with 10% exosome-depleted fetal bovine serum (FBS; Gibco/Thermo Fisher Scientific, 10099141C, Grand Island, NY, USA) and 1% penicillin-streptomycin solution (Gibco/Thermo Fisher Scientific, 15140122, Grand Island, NY, USA) under standard culture conditions (37°C, 5% CO_2_). Cells treated with an equivalent volume of DMSO (<0.1% final concentration) served as vehicle controls. After treatment, conditioned media were collected for exosome isolation and subsequent coculture experiments.

For the downstream assays, HMVECs were used as recipient cells and cocultured with exosomes derived from GW4869-treated or control MCF7/MDA-MB-231 cells. HMVECs were exposed to the exosomes for 48 h before RT-qPCR analysis of mature miR-92b-3p and pri-miR-92b-3p, for 72 h before the CCK-8 proliferation assay, for 48 h before the Transwell migration assay, for 6 d before the tube formation assay, and for 24 h before the transendothelial migration assay, all under standard culture conditions. 

### 2.8 Cell Proliferation Assay

To evaluate endothelial cell proliferation, a Cell Counting Kit-8 (CCK-8) assay (Beyotime, C0037, Shanghai, China) was performed. Briefly, HMVECs were seeded into 96-well plates at a density of 1 × 10^4^ cells/well in 100 μL of medium and pre-incubated at 37°C for 12 h. The cells were then co-cultured with exosomes (5 × 10^11^ particles/mL) derived from MCF-10A cells or varying breast cancer cell models (MCF7-KD, MCF7-WT, MCF7-OE, MDA-MB-231-KD, MDA-MB-231-WT, or MDA-MB-231-OE). At designated time points (0, 12, 24, 48, and 72 h), 10 μL of CCK-8 reagent was added to each well. Following an additional 1 h incubation at 37°C, the absorbance of the samples was measured at 450 nm using a microplate reader (Thermo Fisher Scientific, Multiskan FC, Waltham, MA, USA) to evaluate cell proliferation.

### 2.9 Transwell Experiment

HMVEC migration was evaluated using 24-well Transwell inserts with 8.0 μm pores (Corning Costar, 3422, Corning Inc., Corning, NY, USA). The molecular factor investigated in this assay was exosomal miR-92b-3p. “Different variants of miR-92b-3p” refers to exosomes derived from donor cells with different miR-92b-3p expression levels, including MCF7-KD, MCF7-WT, MCF7-OE, MDA-MB-231-KD, MDA-MB-231-WT, and MDA-MB-231-OE cells. HMVECs were also treated with exosomes from MCF-10A cells as a non-tumor-cell exosome control, and untreated HMVECs served as a blank control. HMVECs were seeded in 6-well plates and co-cultured with 100 μL of exosome suspension (5 × 10^11^ particles/mL; 5 × 10^10^ particles per well) in complete medium containing exosome-depleted FBS at 37°C in a humidified incubator with 5% CO_2_ for 48 h.

After exosome treatment, HMVECs were trypsinized, centrifuged, washed with PBS, and resuspended in serum-free Endothelial Cell Basal Medium-2 (EBM-2; Lonza, CC-3156, Basel, Switzerland) at 1 × 10^6^ cells/mL. A total of 200 μL cell suspension containing 2 × 10^5^ HMVECs was added to the upper chamber of each Transwell insert, and 500 μL complete medium consisting of EBM-2 basal medium supplemented with 10% FBS (Gibco/Thermo Fisher Scientific, 10099141C) was added to the lower chamber as a chemoattractant. After incubation for 24 h at 37°C with 5% CO_2_, non-migrated cells on the upper surface of the membrane were gently removed with cotton swabs. The migrated cells on the lower surface were fixed with methanol (Sinopharm Chemical Reagent Co., Ltd., 10014118, Shanghai, China) for 20 min, stained with crystal violet staining solution (Beyotime Biotechnology, C0121, Shanghai, China) for 40 min, washed three times with distilled water, air-dried, and imaged under a Leica DMi1 microscope (Leica Microsystems GmbH, Wetzlar, Hesse, Germany). Five random fields per insert were photographed and counted for statistical analysis.

### 2.10 Angiogenesis Experiment

For the fibrin gel angiogenesis assay, HMVECs were pretreated for 48 h with exosomes derived from MCF7-WT, MCF7-OE, MDA-MB-231-WT, or MDA-MB-231-OE cells, where OE indicates stable miR-92b-3p overexpression. Exosomes were added at 5 × 10^10^ particles per well in 6-well plates under standard culture conditions at 37°C with 5% CO_2_. After pretreatment, HMVECs were harvested and seeded at 5 × 10^4^ cells per well in 24-well plates. Fibrin gels were prepared using fibrinogen from bovine plasma (2.5 mg/mL; Sigma-Aldrich, F8630, St. Louis, MO, USA), aprotinin as the protease inhibitor (50 μg/mL; Sigma-Aldrich, A1153, St. Louis, MO, USA), and EBM-2 basal medium (Lonza, CC-3156, Basel, Switzerland) supplemented with 2% exosome-depleted FBS (Gibco/Thermo Fisher Scientific, 10099141C), 100 U/mL penicillin, and 100 μg/mL streptomycin. For each well, 225 μL of fibrinogen solution was mixed with bovine thrombin (Sigma-Aldrich, T6884, St. Louis, MO, USA) at a final concentration of 1 U/well and allowed to polymerize at 37°C for 20 min. HMVECs were then suspended in 150 μL fibrinogen-containing medium and seeded onto the gel surface. After incubation for 1 h, exosomes were added at 5 × 10^11^ particles per well, and cells were cultured for 6 days. Angiogenic structures were visualized after incubation with Calcein AM (4 μg/mL; Invitrogen/Thermo Fisher Scientific, C3100MP, Eugene, OR, USA) for 30 min at 37°C.

### 2.11 Transendothelial Migration (Vascular Permeability) Experiment

Transendothelial migration was performed using 24-well Transwell inserts with 8.0 μm pores (Corning Costar, 3422, Corning Inc., Corning, NY, USA). HMVECs were pretreated with exosomes carrying different miR-92b-3p levels for 48 h, harvested, and seeded into the upper chamber at 2 × 10^5^ cells/well in 200 μL complete medium. Endothelial confluence was confirmed before tumor-cell seeding by phase-contrast microscopy, defined as a continuous cobblestone-like monolayer covering ≥95% of the membrane with no visible intercellular gaps. GFP-labeled MCF7 or MDA-MB-231 cells were then added to the upper chamber at 1 × 10^5^ cells/well in serum-free medium (DMEM; Gibco/Thermo Fisher Scientific, C11995500BT), and 600 μL complete medium containing 10% exosome-depleted FBS (Gibco/Thermo Fisher Scientific, 10099141C) was added to the lower chamber. After 12 h, cells were fixed with 4% paraformaldehyde (Beyotime, P0099, Shanghai, China) and imaged using a Leica DMi1 fluorescence microscopy (Leica Microsystems, 11526310). 

### 2.12 Vascular Endothelial Cell Ring Formation Test

To examine the effect of breast cancer cell-derived exosomes on neovascularization, HMVECs were co-cultured with 50 μg/mL of exosomes (Control, MCF7-WT EV+, MCF7-OE EV+, MDA-MB-231-WT EV+, and MDA-MB-231-OE EV+) for 24 h. A 24-well plate was coated with 100 μL Matrigel (Corning, 356234, Corning, NY, USA) per well and incubated at 37°C for 30 min to allow gel formation. Subsequently, the aforementioned co-cultured HMVECs were digested and adjusted to a cell density of 10^6^ cells/mL. A 100 μL cell suspension was then added to the Matrigel-coated 24-well plates. The plates were further incubated in a cell incubator for 5 h, and the number of formed rings was counted using ImageJ software (v. 1.53, National Institutes of Health, Bethesda, MD, USA) for subsequent statistical analysis.

### 2.13 miR-92b-3p Inhibitor Treatment in HMVECs

HMVECs were transfected with 50 nM miR-92b-3p inhibitor (5′-GGAGGCCGGGACGAGUGCAAUA-3′) or inhibitor-NC (GenePharma, B0001, Shanghai, China) using Lipofectamin 3000 (Thermo Fisher Scientific, L3000008). Cells were assigned to four groups: inhibitor-NC, inhibitor, inhibitor + MCF7-Exo, and inhibitor + MDA-MB-231-Exo, with exosomes (1 × 10^8^ particles/mL) added for 48 h. PTEN expression was evaluated by RT-qPCR and Western blotting. RT-qPCR was quantified via the 2^−ΔΔCt^ method using GAPDH as the normalization control. Primers: PTEN (F:5′-TTTGAAGACCATAACCCACCAC-3′, R:5′-ATTACACCAGTTCGTCCCTTTC-3′), GAPDH (F:5′-GGAGCGAGATCCCTCCAAAAT-3′, R:5′-GGCTGTTGTCATACTTCTCATGG-3′). For Western blotting, proteins were separated by 10% SDS-PAGE, transferred to PVDF membranes, and probed with anti-PTEN (Abcam, ab32199, Cambridge, UK, 1:1000) and anti-GAPDH (Sigma-Aldrich, G8795, St. Louis, MO, USA, 1:5000) as the loading control. Protein bands were visualized using an ECL kit (Beyotime, P0018S, Shanghai, China) and quantified by ImageJ software (v. 1.53, National Institutes of Health). 

### 2.14 Tumor Formation Experiments in Nude Mice

A total of 20 female immunodeficient BALB/c nude mice aged 4–6 weeks and weighing 16–20 g were purchased from Shanghai SLAC Laboratory Animal Co., Ltd. (Shanghai, China). All mice were housed under specific-pathogen-free conditions with controlled temperature and humidity, a 12 h light/dark cycle, and free access to standard chow and water. After acclimatization, mice were randomly assigned using a computer-generated randomization method into four experimental groups, with five mice in each group: MCF7-miR-NC, MCF7-miR-92b-3p-OE, MDA-MB-231-miR-NC, and MDA-MB-231-miR-92b-3p-OE. The sample size was determined based on previous xenograft experiments and the principle of minimizing animal use while maintaining sufficient animals for statistical comparison.

For xenograft establishment, mice were subcutaneously injected with 200 μL of sterile saline containing control or miR-92b-3p-overexpressing breast cancer cells at a concentration of 2 × 10^7^ cells/mL. For the MCF7 xenograft models, 17β-estradiol pellets (0.72 mg, 60-day release; Innovative Research of America, SE-121, Sarasota, FL, USA) were subcutaneously implanted before tumor cell inoculation. Tumor length and width were measured with calipers, and tumor volume was calculated using the formula: volume = length × width^2^/2. The primary outcome measures were tumor volume and tumor weight, and secondary outcome measures included miR-92b-3p expression, tumor vascularization, and circulating tumor cell counts. Investigators responsible for tumor measurement and outcome assessment were blinded to group allocation.

Mice were monitored regularly for general health status, body weight, tumor growth, and signs of distress. Humane endpoints included severe weight loss, impaired mobility, tumor ulceration, or excessive tumor burden. No animals were excluded from the final analysis. At 21 days after tumor cell inoculation, mice were euthanized according to approved institutional animal care procedures, and xenograft tumors and peripheral blood samples were collected for subsequent analyses. All animal procedures were conducted in accordance with the ARRIVE Essential 10 guidelines and were approved by the Ethics Committee of Experimental Animal Center of Jilin University (Approval No. 2023-1206).

### 2.15 Flow Cytometry for Circulating Tumor Cells

To quantify circulating tumor cells (CTCs) *in vivo*, peripheral blood samples were collected from tumor-bearing mice at the endpoint of the xenograft experiment on day 21 and placed into EDTA-coated anticoagulant tubes. For each sample, 50 μL of well-mixed anticoagulated whole blood was transferred into a flow cytometry tube. Red blood cells were lysed by adding 1 mL of red blood cell lysis buffer (Beyotime Biotechnology, C3702, Shanghai, China) and incubating for 5 min at room temperature in the dark. The reaction was terminated by adding 2 mL of PBS, followed by centrifugation at 400× *g* for 5 min at 4°C. The supernatant was carefully discarded, and the cell pellet was washed twice with PBS by centrifugation at 400× *g* for 5 min at 4°C. The final cell pellet was resuspended in 300 μL PBS and filtered through a 40 μm cell strainer before flow cytometric analysis.

Because the inoculated breast cancer cells, including MCF7 and MDA-MB-231 cells, stably expressed green fluorescent protein (GFP), CTCs were identified as GFP-positive events without additional antibody staining. GFP-positive MCF7 or MDA-MB-231 cells were used as positive controls, and blood samples from non-tumor-bearing mice were used as negative controls to define the GFP-positive gate. Data were acquired using an Attune CytPix Flow Cytometer (Thermo Fisher Scientific, Waltham, MA, USA) in the FITC channel. During analysis, debris was excluded based on forward scatter and side scatter parameters, and single cells were selected using FSC-A/FSC-H gating. GFP-positive events were then quantified as CTCs. The number of CTCs was expressed as GFP-positive cells per 50 μL of peripheral blood.

### 2.16 Immunofluorescence Staining

Tissues from mice were dehydrated and cut into 2 μm thick sections, which were subsequently rinsed in 0.01M PBS three times for 5 min each. Following this, the sections were treated with 10% normal goat serum (Beyotime Biotechnology, C0265, Shanghai, China) at 37°C for 45 min. CD31 (1:100) (Abcam, ab28364, Cambridge, UK) was then added and incubated overnight at 4°C. Then, the sections were washed three times in PBS for 5 min per wash. Subsequently, goat anti-rabbit IgG-HL (1:500) (Abcam, ab6721, Cambridge, MA, USA) was applied and incubated for 1 h at room temperature. After three additional washes with PBS for 5 min each, the tissue sections were mounted on slides, counterstained with DAPI (Beyotime Biotechnology, C1002) for 10 min at room temperature, sealed, and observed under a microscope (Leica Microsystems, 11526310) for photographic documentation. 

### 2.17 Western Blotting

Total protein from cells or exosomes was extracted using RIPA lysis buffer (Beyotime, P0013B, Shanghai, China) supplemented with protease and phosphatase inhibitors (Yeasen Biotechnology, 20124ES03). Protein concentrations were determined using a BCA Protein Assay Kit (Beyotime, P0012, Shanghai, China). Equal amounts of protein (10 μg/lane) were separated by 12% SDS-PAGE and transferred onto PVDF membranes (Millipore, IPVH00010, Burlington, MA, USA). The membranes were blocked with 5% non-fat milk at 37°C for 2 h and incubated overnight at 4°C with primary antibodies against CD9 (Abcam, ab236630, 1:1000), CD63 (Abcam, ab134045, 1:1000), TSG101 (Abcam, ab125011, 1:1000), Calnexin (Abcam, ab22595, 1:1000), Cytochrome C (Abcam, ab133504, 1:1000), PTEN (Abcam, ab32199, 1:1000), VE-cadherin (Abcam, ab33168, 1:1000), β-actin (Sigma-Aldrich, A1978, St. Louis, MO, USA, 1:5000), and GAPDH (Sigma-Aldrich, G8795, 1:5000). After TBST washing, membranes were incubated for 2 h at room temperature with either HRP-linked anti-rabbit IgG secondary antibody (Cell Signaling Technology, 7074, Danvers, MA, USA, 1:2000) or HRP-linked anti-mouse IgG secondary antibody (Cell Signaling Technology, 7076, Danvers, MA, USA, 1:2000), according to the host species of the corresponding primary antibodies. Bands were visualized using an enhanced ECL chemiluminescence kit (Beyotime, P0018S, Shanghai, China) and quantified via ImageJ software (v. 1.53, National Institutes of Health).

### 2.18 Statistical Analysis

Data analysis was performed using R and SPSS statistical software. Unless otherwise indicated, cell-based experiments were repeated independently at least three times. Quantitative data are presented as mean ± standard deviation (x ± s). Data distribution was assessed before parametric testing; comparisons between two groups were performed using unpaired Student’s *t* test for normally distributed data or Mann-Whitney U test for non-normally distributed data. Comparisons among multiple groups were analyzed by one-way ANOVA followed by Tukey’s post hoc test when parametric assumptions were met; otherwise, the Kruskal-Wallis test followed by Dunn’s multiple-comparison test was used. For TCGA differential expression analysis, multiple testing was controlled by Benjamini-Hochberg FDR adjustment. Receiver operating characteristic (ROC) analysis for plasma exosomal miR-92b-3p was performed using a binary logistic regression model to distinguish breast cancer cases from non-cancer controls, and the area under the curve (AUC) with 95% confidence interval was estimated by the DeLong method. The optimal cutoff value was determined by the maximum Youden index. Survival curves were compared by the log-rank test. All tests were two-sided, and *p* < 0.05 was considered statistically significant.

## 3 Results

### 3.1 Exosomal miR-92b-3p Expression Level Was Significantly Increased in Blood and Tissue Samples from Breast Cancer Patients

Differential miRNA expression was first screened in the TCGA-BRCA cohort. Analysis of 1200 tumor samples and 110 normal breast tissues identified 510 differentially expressed miRNAs under the preset criteria (FDR < 0.05, Log2 |FC| > 1), of which 200 were upregulated and 310 were downregulated. Among them, miR-92b-3p was significantly increased in breast cancer tissues (Fig. S1). When pathological stage was analyzed according to the TCGA annotations, miR-92b-3p showed an overall tendency to increase from stage I to stage IV, and the exploratory grouped comparison of stage I–III versus stage IV further suggested higher expression in stage IV disease (Fig. S2). Survival analysis using the median expression value as the cutoff indicated that patients with high miR-92b-3p expression had shorter overall survival than those with low expression (Fig. S3).

**Figure 1:**
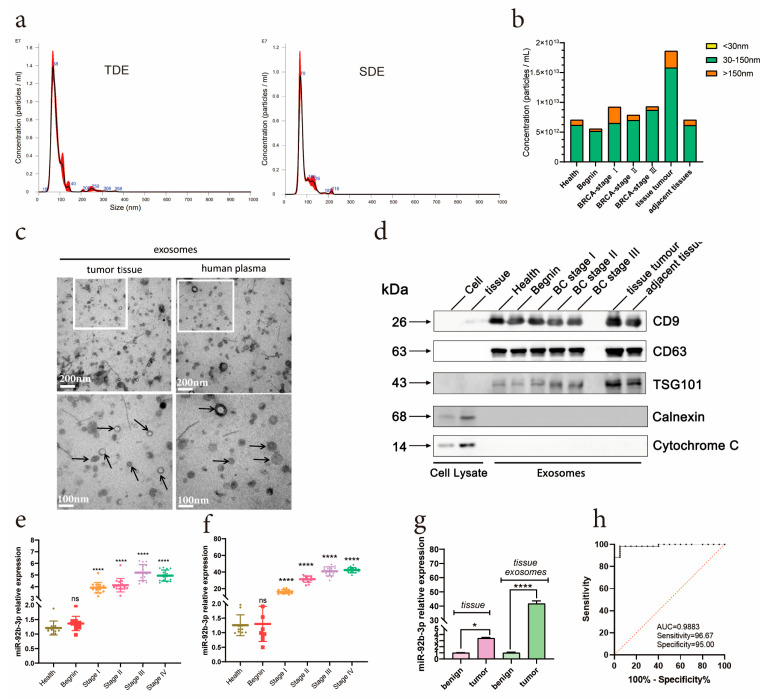
miR-92b-3p is elevated in blood and tissue samples from patients with breast cancer. (**a**) Representative nanoparticle tracking analysis (NTA) profiles showing the size distribution of plasma- and tissue-derived exosome-enriched vesicles. (**b**) Quantification of vesicle concentration by NTA. (**c**) Representative transmission electron microscopy (TEM) images of exosome-enriched vesicles (arrows). (**d**) Representative Western blot analysis of vesicle-associated markers CD9, CD63, and TSG101 and negative markers Calnexin and Cytochrome C. (**e**) Relative miR-92b-3p expression in plasma from healthy controls (n = 10), patients with benign breast disease (n = 10), and patients with breast cancer stratified by clinical stage I-IV (n = 15 per stage). The relative expression levels were normalized to the internal reference U6 using the 2^−ΔΔCt^ method. (**f**) Relative miR-92b-3p expression in plasma-derived exosome-enriched vesicles from the same cohort. (**g**) Relative miR-92b-3p expression in paired tumor and paracancerous tissues and the corresponding tissue-derived exosome-enriched vesicles from patients with breast cancer (n = 10 pairs). The data in (**f**,**g**) were similarly normalized to U6. (**h**) Receiver operating characteristic (ROC) curve of plasma exosomal miR-92b-3p for distinguishing breast cancer cases (n = 60) from non-cancer controls (n = 20). Data in quantified panels are presented as mean ± SD. Representative images/blots are shown from three independent preparations. Multiple-group comparisons were analyzed by one-way ANOVA with Tukey’s post hoc test, and two-group comparisons were analyzed by two-tailed Student’s *t* test as appropriate. Scale bars are indicated in the panels. n.s., not significant; *p < 0.05; ****p < 0.0001.

Next, exosome-enriched vesicles were isolated from plasma and tissue samples and characterized by NTA, TEM, and Western blotting (Fig. 1a–d). In the clinical cohort comprising 60 breast cancer patients, 10 benign breast disease patients, and 10 healthy controls, RT-qPCR showed that miR-92b-3p levels were significantly elevated in both plasma and plasma-derived exosomes from breast cancer patients, with higher values observed in more advanced clinical stages (Fig. 1e,f). Consistently, miR-92b-3p expression in tumor tissues was 4.2-fold higher than in paired paracancerous tissues, and tissue-derived exosomes from tumor samples also showed markedly increased miR-92b-3p levels (Fig. 1g). These findings support a clinical association between elevated miR-92b-3p and breast cancer burden in this cohort.

To further assess discriminatory performance, plasma exosomal miR-92b-3p was entered into a binary logistic regression model contrasting breast cancer cases with non-cancer controls (benign breast disease plus healthy individuals). ROC analysis yielded an AUC of 0.9883 (95% CI: 0.9651–1.0000), with 96.67% sensitivity and 95.00% specificity at the optimal Youden-derived cutoff (Fig. 1h). Given the modest sample size and the absence of an external validation cohort, these results are best interpreted as preliminary evidence that plasma exosomal miR-92b-3p may serve as an adjunctive discriminatory marker rather than a stand-alone diagnostic test.

### 3.2 Exosomes Derived from Breast Cancer Cells Delivered miR-92b-3p into Endothelial Cells

**Figure 2:**
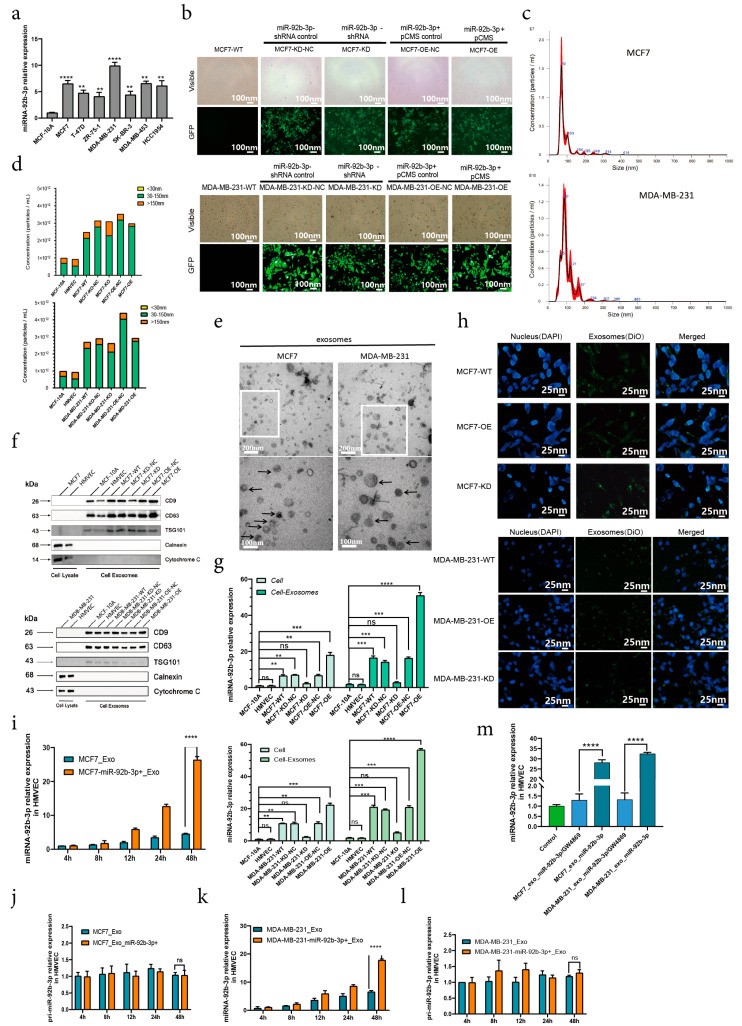
Exosomes derived from breast cancer cells delivered miR-92b-3p into endothelial cells. (**a**) Relative miR-92b-3p expression in breast cancer cell lines and MCF-10A cells. (**b**) Representative fluorescence images of stable miR-92b-3p-overexpressing and miR-92b-3p-knockdown cell models. (**c**) Representative NTA profiles of cell-derived exosome-enriched vesicles. (**d**) Quantification of vesicle concentration by NTA. (**e**) Representative TEM images of exosome-enriched vesicles (arrows). (**f**) Representative Western blot analysis of vesicle-associated markers. (**g**) Relative miR-92b-3p expression in donor cells and their released exosome-enriched vesicles. (**h**) Representative fluorescence images showing uptake of DiO-labeled breast cancer cell-derived exosomes by HMVECs. (**i**) Relative expression of mature miR-92b-3p and (**j**) pri-miR-92b-3p in recipient HMVECs following co-culture with MCF7-derived exosomes (dose: 5 × 10^11^ particles/mL) over a time course of 4, 8, 12, 24, and 48 h. (**k**) Relative expression of mature miR-92b-3p and (**l**) pri-miR-92b-3p in HMVECs following co-culture with MDA-MB-231-derived exosomes (dose: 5 × 10^11^ particles/mL) over the same 4 to 48 h time course. (**m**) Relative miR-92b-3p expression in HMVECs after GW4869 treatment. Quantified data are presented as mean ± SD from three independent biological replicates (n = 3). Representative images and blots are shown from three independent experiments. Multiple-group comparisons were analyzed by one-way ANOVA with Tukey’s post hoc test. Scale bars are indicated in the panels. n.s., not significant; **p < 0.01, ***p < 0.001, ****p < 0.0001.

Three estrogen receptor (ER)-positive cell lines (MCF7, T-47D, and ZR-75-1), four ER-negative cell lines (MDA-MB-231, SK-BR-3, MDA-MB-453, and HCC1954), and MCF-10A cells were screened for miR-92b-3p expression. miR-92b-3p was significantly upregulated in all seven breast cancer cell lines compared with MCF-10A, with the highest expression observed in MCF7 among ER-positive cells and in MDA-MB-231 among ER-negative cells (Fig. 2a). These two lines, representing distinct molecular backgrounds, were therefore selected for subsequent experiments. Stable miR-92b-3p-overexpressing cells (MCF7-OE and MDA-MB-231-OE) and miR-92b-3p-knockdown cells (MCF7-KD and MDA-MB-231-KD) were then established. Fluorescence imaging confirmed successful construction of the corresponding cell models (Fig. 2b).

After exosomes from MCF-10A, HMVEC, MCF7, and MDA-MB-231 cells were verified by NTA, TEM, and marker analysis (Fig. 2c–f), RT-qPCR showed that miR-92b-3p was enriched in exosomes released by breast cancer cells, particularly in the MCF7-OE group (Fig. 2g). DiO-labeling experiments confirmed efficient uptake of breast cancer cell-derived exosomes by HMVECs (Fig. 2h). Following 48 h coculture with exosomes from MCF7-OE or MDA-MB-231-OE cells, mature miR-92b-3p in HMVECs increased by approximately sevenfold, whereas pri-miR-92b-3p showed no significant change (Fig. 2i–l), supporting transfer of exogenous miR-92b-3p rather than induction of its endogenous transcription. In the GW4869-treated coculture system, miR-92b-3p accumulation in HMVECs was significantly reduced (Fig. 2m), supporting an exosome-dependent component in this intercellular transfer process rather than proving that miR-92b-3p is the only vesicle-associated effector.

### 3.3 Breast Cancer Cell-Derived Exosomal miR-92b-3p Promoted Proliferation, Migration, Tube-Forming and Permeability of Vascular Endothelial Cells

**Figure 3:**
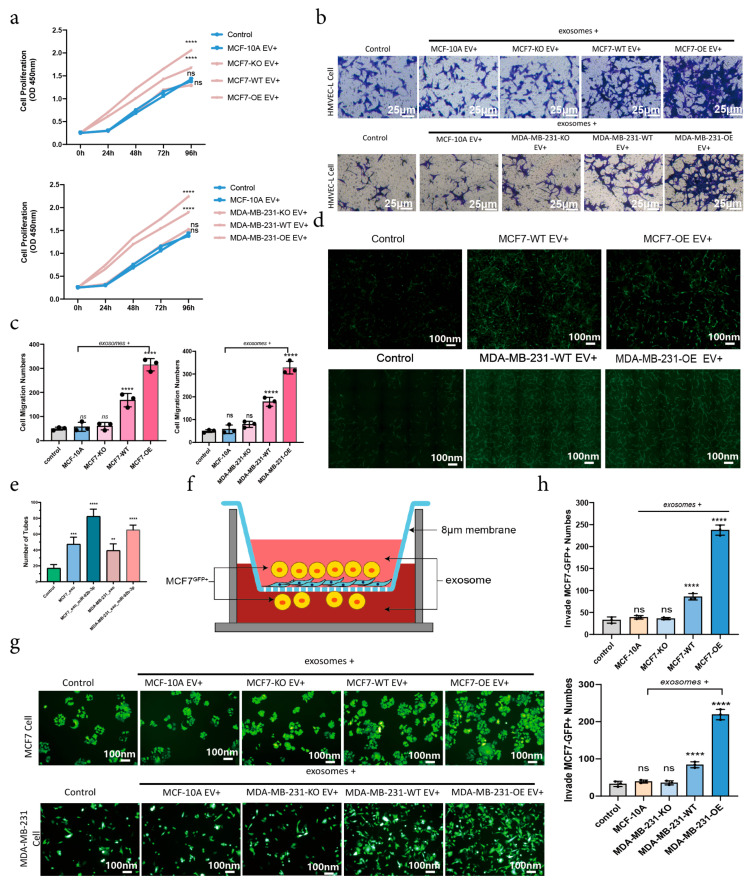
Exosomal miR-92b-3p from breast cancer cells enhanced endothelial proliferation, migration, tube formation, and permeability. (**a**) Proliferation of HMVECs after coculture with exosome-enriched vesicles derived from control cells, MCF7-KD, MDA-MB-231-KD, MCF7-WT, MDA-MB-231-WT, MCF7-OE, or MDA-MB-231-OE cells. (**b**) Representative images of HMVEC migration in Transwell assays. (**c**) Quantification of migrated HMVECs. (**d**) Representative images of endothelial tube/ring-like structure formation after exosome treatment. (**e**) Quantification of angiogenic structure formation. (**f**) Schematic illustration of the transendothelial migration assay. (**g**) Representative images of GFP-labeled breast cancer cells crossing the endothelial monolayer after HMVEC pretreatment with exosomes carrying different levels of miR-92b-3p. (**h**) Quantification of transendothelial tumor cell migration. Data are presented as mean ± SD from three independent biological replicates (n = 3). Representative images are shown from three independent experiments. Multiple-group comparisons were analyzed by one-way ANOVA with Tukey’s post hoc test. Scale bars are indicated in the panels. ns not significant; **p < 0.01, ***p < 0.001, ****p < 0.0001.

To examine whether exosomal miR-92b-3p alters endothelial behavior, HMVECs were cocultured for 72 h with exosomes derived from control cells, MCF7-KD, MDA-MB-231-KD, MCF7-WT, MDA-MB-231-WT, MCF7-OE, or MDA-MB-231-OE cells. HMVEC proliferation was significantly increased after treatment with MCF7-WT EV+ and MDA-MB-231-WT EV+ compared with control and MCF-10A EV+ groups, and this effect was further enhanced by MCF7-OE EV+ and MDA-MB-231-OE EV+ exosomes. Conversely, exosomes from the corresponding knockdown groups attenuated endothelial proliferation relative to their WT counterparts (Fig. 3a). Transwell assays showed a similar pattern for endothelial migration, with miR-92b-3p-high exosomes increasing the number of migrating HMVECs, whereas miR-92b-3p knockdown reduced this effect (Fig. 3b,c). In matrix-based angiogenesis assays, exosomes with higher miR-92b-3p content also promoted tube- or ring-like structure formation in HMVECs, and the extent of angiogenesis was positively associated with miR-92b-3p abundance in donor-cell exosomes (Fig. 3d,e).

Because metastatic dissemination depends not only on endothelial growth and migration but also on endothelial barrier integrity, we next examined permeability-related transendothelial tumor cell migration. HMVECs were first pretreated with exosomes carrying different levels of miR-92b-3p and then used to form a confluent endothelial monolayer on Transwell inserts (Fig. 3f). Under these conditions, GFP-labeled MCF7 and MDA-MB-231 cells crossed the endothelial layer more efficiently when HMVECs had been exposed to miR-92b-3p-high exosomes, whereas reduced transfer of miR-92b-3p attenuated this phenotype (Fig. 3g,h). These data indicate that breast cancer-derived exosomal miR-92b-3p promotes endothelial activation and barrier loosening, thereby facilitating transendothelial passage of tumor cells.

### 3.4 Overexpression of miR-92b-3p Promoted Tumor Angiogenesis and Dissemination In Vivo

**Figure 4:**
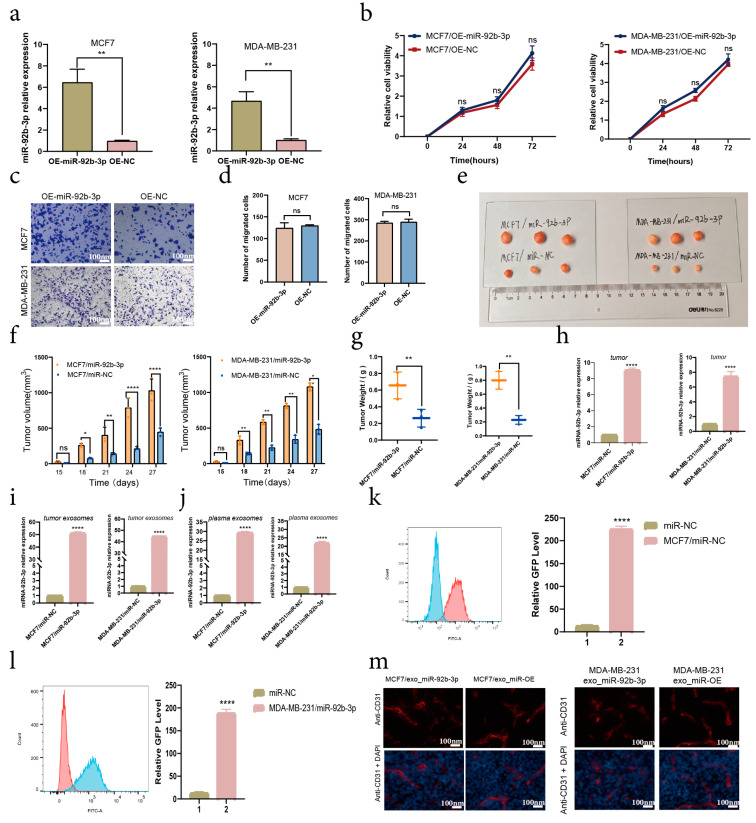
Overexpression of miR-92b-3p promoted tumor angiogenesis *in vivo*. (**a**) Relative miR-92b-3p expression in breast cancer cells after transfection. (**b**) *In vitro* proliferation of breast cancer cells with or without miR-92b-3p overexpression. (**c**) Representative images of tumor cell migration assays. (**d**) Quantification of migrated tumor cells. (**e**) Representative images of xenograft tumors at endpoint. (**f**) Tumor growth curves. (**g**) Tumor weights at sacrifice. (**h**) Relative miR-92b-3p expression in xenograft tumor tissues from mice inoculated with control (miR-NC) or miR-92b-3p-overexpressing breast cancer cells (MCF7 and MDA-MB-231). (**i**) Relative miR-92b-3p expression in tumor-derived exosomes isolated from the corresponding xenografts. (**j**) Relative miR-92b-3p expression in plasma-derived exosomes collected from the tumor-bearing mice. (**k**) Representative flow cytometry plots of GFP-positive circulating tumor cells (CTCs) in peripheral blood. (**l**) Quantification of GFP-positive CTCs. (**m**) Representative CD31 immunofluorescence images of xenograft tissues. Data are presented as mean ± SD; n = 3 independent biological replicates for panels (**a**–**d**) and n = 5 mice per group for panels (**e**–**m**). Representative images are shown from independent experiments or individual mice as indicated. Multiple-group comparisons were analyzed by one-way ANOVA with Tukey’s post hoc test, and two-group comparisons were analyzed by two-tailed Student’s *t* test as appropriate. Scale bars are indicated in the panels. n.s., not significant; *p < 0.05; **p < 0.01; ****p < 0.0001.

To determine whether miR-92b-3p exerts direct effects on tumor cells or primarily influences tumor behavior through the microenvironment, we first evaluated its overexpression in breast cancer cell lines (Fig. 4a). *In vitro*, miR-92b-3p overexpression did not significantly alter the proliferation or migration of breast cancer cells (Fig. 4b–d). By contrast, in the subcutaneous mouse model, tumors formed by miR-92b-3p-overexpressing cells grew larger and weighed more than controls after 21 days (Fig. 4e–g). miR-92b-3p levels were also markedly increased in xenograft tissues and in plasma-derived exosomes from tumor-bearing mice, with the strongest increase detected in tumor tissue-derived exosomes (Fig. 4h–j). These findings suggest that the *in vivo* phenotype is more consistent with microenvironment-mediated effects than with a strong tumor cell-intrinsic proliferative program.

Circulating tumor cells (CTCs) were then quantified by flow cytometry as an *in vivo* readout of tumor cell dissemination. Mice bearing miR-92b-3p-overexpressing tumors showed a higher number of GFP-positive CTCs in peripheral blood than control animals (Fig. 4k,l). Consistent with this finding, CD31 immunofluorescence demonstrated increased tumor vascularization in the miR-92b-3p-overexpression groups in both the MCF7 and MDA-MB-231 models (Fig. 4m). Together, these data support a role for miR-92b-3p in promoting tumor angiogenesis and early vascular dissemination *in vivo*.

### 3.5 PTEN Was Targeted and Negatively Regulated by miR-92b-3p

**Figure 5:**
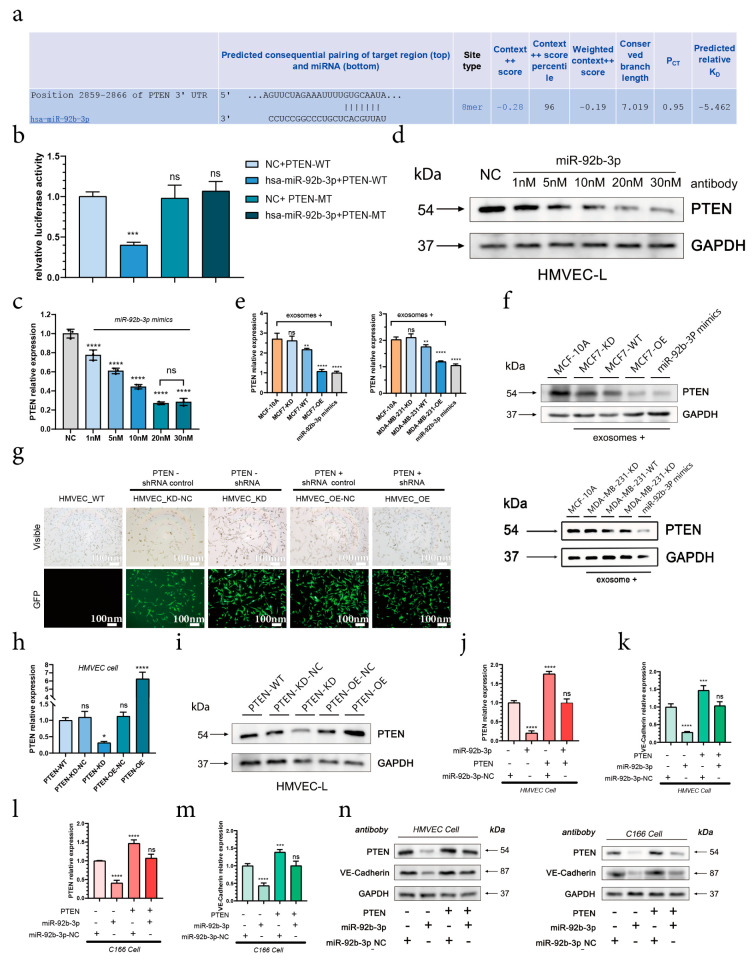
PTEN is a direct and functionally relevant endothelial target of miR-92b-3p. (**a**) Predicted binding site between miR-92b-3p and the PTEN 3′UTR. (**b**) Dual-luciferase reporter assay showing the effect of miR-92b-3p on wild-type and mutant PTEN reporters. (**c**) Relative PTEN mRNA expression in HMVECs after transfection with different concentrations of miR-92b-3p mimics. (**d**) Representative Western blot and quantification of PTEN protein expression under the same conditions. (**e**) Relative PTEN mRNA expression in HMVECs after treatment with exosomes carrying different levels of miR-92b-3p. (**f**) Representative Western blot and quantification of PTEN protein expression after exosome treatment. (**g**) Representative fluorescence images of stable HMVEC lines with altered PTEN expression. (**h**) Relative PTEN mRNA expression in stable PTEN-knockdown (PTEN-KD) or PTEN-overexpressing (PTEN-OE) HMVEC lines and their respective controls. (**i**) Representative Western blot demonstrating PTEN protein expression in the established PTEN-altered HMVEC models. (**j**) Relative mRNA expression of PTEN and (**k**) VE-Cadherin in HMVECs following co-culture with miR-92b-3p-altered exosomes, with or without PTEN rescue. (**l**) Relative mRNA expression of PTEN and (**m**) VE-Cadherin in murine C166 endothelial cells under the same experimental conditions. (**n**) Representative Western blots evaluating PTEN and VE-Cadherin protein levels in both HMVECs and C166 cells across the different corresponding treatment groups. Data are presented as mean ± SD from three independent biological replicates (n = 3). Representative images and blots are shown from three independent experiments. Multiple-group comparisons were analyzed by one-way ANOVA with Tukey’s post hoc test. Scale bars are indicated in the panels. n.s., not significant; *p < 0.05; **p < 0.01; ***p < 0.001; ****p < 0.0001.

To investigate the molecular basis of the endothelial phenotype, TargetScan analysis identified PTEN as a candidate target of miR-92b-3p (Fig. 5a). Dual-luciferase reporter assays showed that miR-92b-3p significantly suppressed the activity of the PTEN wild-type reporter but not the mutant construct, confirming direct binding to the predicted PTEN 3′UTR site (Fig. 5b). In HMVECs, transfection with miR-92b-3p mimics led to a dose-dependent reduction in PTEN mRNA and protein expression (Fig. 5c,d). Similarly, exosomes from MCF7-OE and MDA-MB-231-OE cells reduced PTEN expression in recipient HMVECs, whereas miR-92b-3p mimic treatment produced a comparable inhibitory effect (Fig. 5e,f). These results identify PTEN as a direct and functionally relevant endothelial target of miR-92b-3p in this model.

To further examine whether PTEN-associated endothelial changes were reproducible across endothelial backgrounds, we established PTEN-overexpressing and PTEN-knockdown HMVEC models (Fig. 5g–i) and additionally assessed both HMVECs and murine C166 endothelial cells after coculture with exosomes containing different levels of miR-92b-3p. In both endothelial models, higher exosomal miR-92b-3p was associated with lower PTEN expression and concomitant downregulation of VE-cadherin, whereas PTEN overexpression partially restored VE-cadherin levels (Fig. 5j–n). These observations support PTEN as a key mediator linking exosomal miR-92b-3p to endothelial junction remodeling, while also indicating that the directional effect is reproducible in more than one endothelial cell context.

### 3.6 The miR-92b-3p/PTEN Pathway Mediated Cancer Cell Invasion and Endothelial Cell Angiogenesis

**Figure 6:**
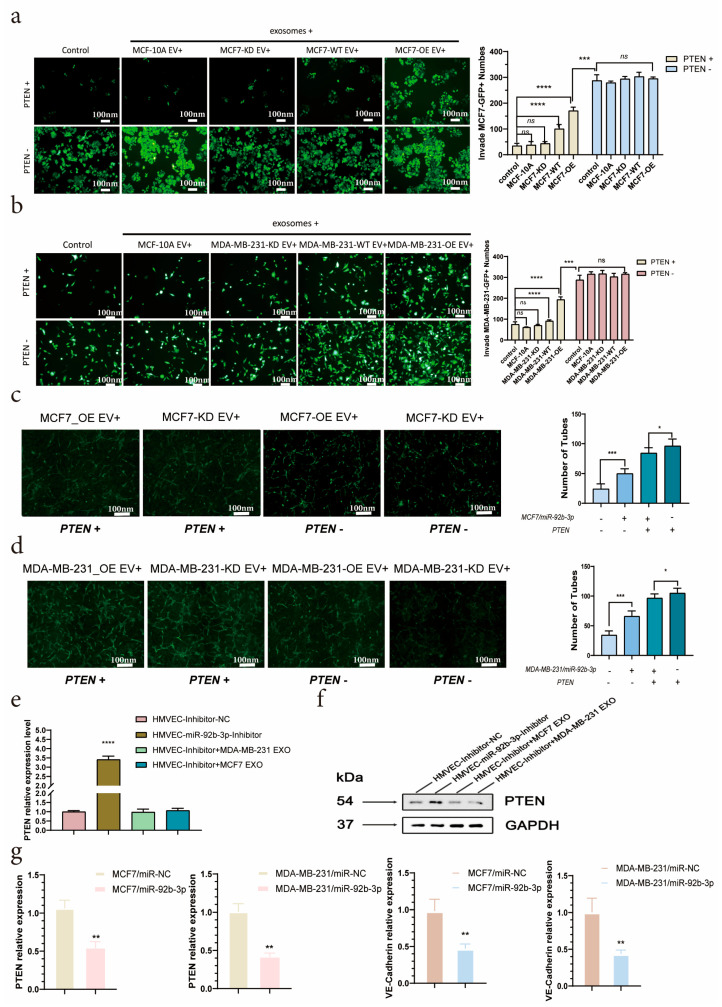
PTEN functionally mediates the effects of exosomal miR-92b-3p on endothelial permeability and angiogenesis. (**a**) Representative images and quantification of GFP-labeled MCF7 cells crossing HMVEC monolayers. HMVECs were pre-treated with exosomes (5 × 10^11^ particles/mL) derived from MCF7 cells with varying miR-92b-3p expression: MCF7-KD (knockdown), MCF7-WT (wild-type), and MCF7-OE (overexpression). The HMVEC monolayers were established under different PTEN expression backgrounds (PTEN+: PTEN overexpressing cells; PTEN−: PTEN knockdown cells). (**b**) Representative images and quantification of GFP-labeled MDA-MB-231 cells crossing HMVEC monolayers. HMVECs were pre-treated with exosomes (5 × 10^11^ particles/mL) derived from MDA-MB-231 cells with varying miR-92b-3p expression: MDA-MB-231-KD (knockdown), MDA-MB-231-WT (wild-type), and MDA-MB-231-OE (overexpression). The HMVEC monolayers were established under different PTEN expression backgrounds (PTEN+: PTEN overexpressing cells; PTEN−: PTEN knockdown cells). (**c**) Representative images and quantification of endothelial ring/tube formation in HMVECs with different PTEN expression levels after treatment with exosomes from MCF7 cells expressing different levels of miR-92b-3p. (**d**) Representative images and quantification of endothelial ring/tube formation after treatment with exosomes from MDA-MB-231 cells expressing different levels of miR-92b-3p. (**e**) Relative PTEN mRNA expression in HMVECs transfected with miR-92b-3p inhibitor and then exposed to breast cancer cell-derived exosomes. (**f**) Representative Western blot of PTEN protein expression under the same conditions. (**g**) Relative PTEN and VE-cadherin expression in xenograft tissues from control and miR-92b-3p-overexpressing groups. Data are presented as mean ± SD; n = 3 independent biological replicates for panels (**a**–**f**) and n = 5 mice per group for panel (**g**). Representative images and blots are shown from independent experiments. Multiple-group comparisons were analyzed by one-way ANOVA with Tukey’s post hoc test. Scale bars are indicated in the panels. n.s., not significant; *p < 0.05; **p < 0.01; ***p < 0.001; ****p < 0.0001.

To test whether PTEN mediates the permeability-related effects of exosomal miR-92b-3p, transendothelial migration assays were performed using HMVECs with PTEN overexpression or PTEN knockdown together with exosomes carrying different amounts of miR-92b-3p. PTEN overexpression attenuated the transendothelial migration of GFP-labeled MCF7 and MDA-MB-231 cells, whereas PTEN knockdown enhanced tumor-cell passage across the endothelial monolayer. Within this framework, exosomes enriched in miR-92b-3p further increased migration, and the effect generally paralleled miR-92b-3p abundance in donor-cell exosomes (Fig. 6a,b). In matrix-based angiogenesis assays, PTEN knockdown favored endothelial ring formation, while PTEN overexpression suppressed the pro-angiogenic effect of miR-92b-3p-high exosomes (Fig. 6c,d). These findings support PTEN as a major functional mediator of the endothelial permeability and angiogenic phenotypes induced by breast cancer-derived exosomal miR-92b-3p.

To further evaluate the specificity of this interaction, HMVECs were transfected with a miR-92b-3p inhibitor before exposure to exosomes from MCF7 or MDA-MB-231 cells. Compared with the inhibitor negative control, direct inhibition of miR-92b-3p increased PTEN expression in HMVECs, whereas subsequent coculture with breast cancer cell-derived exosomes blunted this increase (Fig. 6e,f), consistent with transfer of miR-92b-3p from donor cells. In tumor tissues from the mouse model, miR-92b-3p overexpression was likewise accompanied by reduced PTEN and VE-cadherin expression (Fig. 6g). Together, these data support a functional contribution of the miR-92b-3p/PTEN axis to endothelial remodeling in breast cancer, while not excluding the possibility that additional downstream targets or signaling pathways are also involved.

## 4 Discussion

Metastatic progression in breast cancer is shaped not only by the intrinsic properties of tumor cells, but also by changes in the vascular microenvironment that support angiogenesis, endothelial remodeling, and tumor cell entry into the circulation. In this study, we integrated TCGA analysis, patient-derived samples, endothelial functional assays, and xenograft experiments to examine the role of breast cancer-derived exosomal miR-92b-3p in this process. The results support three related observations. miR-92b-3p was increased in the TCGA-BRCA cohort and in plasma, plasma-derived exosome-enriched vesicles, tumor tissues, and tissue-derived exosome-enriched vesicles from patients with breast cancer. Breast cancer cell-derived exosomes were then shown to transfer miR-92b-3p into endothelial cells, where they enhanced migration, angiogenic activity, and permeability-related transendothelial tumor cell passage. At the mechanistic level, PTEN was confirmed as a direct target of miR-92b-3p in endothelial cells, and PTEN rescue experiments supported its functional importance in the vascular phenotype observed here. Notably, miR-92b-3p overexpression had little effect on breast cancer cell proliferation or migration *in vitro*, yet promoted tumor growth, vascularization, and circulating tumor cell release *in vivo*. Taken together, these findings argue that, in this setting, the major effect of miR-92b-3p is exerted through the tumor microenvironment, particularly the endothelium, rather than through a dominant tumor cell-intrinsic program.

The clinical part of the study suggests that miR-92b-3p may have value as a circulating marker, but this point needs to be interpreted with appropriate caution. Circulating and exosomal miRNAs have attracted considerable interest as minimally invasive indicators of tumor burden and disease behavior [21]. Du et al. previously reported that serum miR-92b-3p is elevated in breast cancer and is associated with adverse clinicopathological features [17]. Our results are in line with that report and extend it by showing that miR-92b-3p is enriched not only in plasma, but also in plasma-derived exosome-enriched vesicles, with levels tending to increase with clinical stage. This pattern is consistent with a link between circulating miR-92b-3p and dissemination-related biology. At the same time, the diagnostic signal should not be overstated. Although the ROC analysis yielded a high AUC in the present cohort, the sample size was limited, the study was conducted at a single center, and no external validation cohort was available. For now, plasma exosomal miR-92b-3p is better regarded as an adjunctive discriminatory marker than as a stand-alone diagnostic test. Similarly, the prognostic implication of miR-92b-3p in the present work is limited to the TCGA-based survival association and was not independently validated in our clinical cohort.

A notable aspect of the present work is that it places miR-92b-3p in an endothelial and exosomal context. Previous studies on miR-92b-3p have largely focused on tumor cell-intrinsic signaling in other cancer types [13,14,17], whereas our data indicate that exosome-mediated transfer to vascular endothelial cells is a major part of its biological activity in breast cancer. After coculture with breast cancer-derived exosomes, HMVECs showed a clear increase in mature miR-92b-3p without a corresponding rise in pri-miR-92b-3p, favoring direct intercellular transfer over endogenous transcriptional induction. The GW4869 experiment further supported an exosome-dependent component in this process. Still, that result should not be read too broadly. GW4869 does not establish that miR-92b-3p is the only active cargo involved, and tumor-derived exosomes are known to carry multiple proteins, lipids, and nucleic acids that can influence endothelial behavior in parallel [7,8,9,10,11,22]. Our data therefore support exosomal miR-92b-3p as an important functional effector within a broader vesicle-mediated signaling network, rather than as the sole determinant of the phenotype.

The use of both MCF7 and MDA-MB-231 cells also adds context to the interpretation of these findings. These lines represent distinct molecular backgrounds within breast cancer, with MCF7 modeling a hormone receptor-positive, less invasive phenotype and MDA-MB-231 representing a more aggressive receptor-negative phenotype [4,23,24]. miR-92b-3p was elevated in both settings, and exosomes from both cell lines produced similar endothelial effects, suggesting that the miR-92b-3p/PTEN axis is not confined to a single subtype background. This does not mean that the pathway is biologically identical across all forms of breast cancer. Given the heterogeneity of the disease, the magnitude and clinical relevance of this axis may still depend on hormone receptor status, HER2 expression, broader genomic context, or prior treatment exposure. Because clinicopathological annotation in the present cohort was incomplete, we were not able to perform a meaningful subtype-stratified clinical analysis. Larger and better-annotated cohorts will be needed to define this point more clearly.

Mechanistically, the present study identifies PTEN as a direct and functionally relevant endothelial target of miR-92b-3p. PTEN is a well-established negative regulator of PI3K/AKT signaling and has recognized roles in survival, migration, angiogenesis, and endothelial junction stability [25,26]. Here, dual-luciferase assays confirmed direct binding of miR-92b-3p to the PTEN 3′UTR, and both miR-92b-3p mimics and breast cancer-derived exosomes reduced PTEN expression in recipient endothelial cells. Functionally, PTEN knockdown enhanced endothelial angiogenesis and transendothelial tumor cell migration, whereas PTEN overexpression attenuated these effects. These findings place PTEN at the center of the endothelial response described in this study. However, the data do not support the conclusion that PTEN is the exclusive target through which miR-92b-3p acts. Like most miRNAs, miR-92b-3p is likely to regulate multiple transcripts simultaneously, and the phenotype observed here probably reflects PTEN-centered signaling together with additional targets that remain to be defined.

The link between PTEN suppression and endothelial barrier remodeling is particularly relevant to the biological interpretation of our results. In both HMVECs and C166 endothelial cells, higher exosomal miR-92b-3p was associated with reduced PTEN expression and concomitant downregulation of VE-cadherin. VE-cadherin is a key component of endothelial adherens junctions and an important determinant of vascular barrier integrity; its reduction would be expected to favor vascular leakiness and facilitate tumor cell passage across the endothelial lining. Although the downstream pathway was not directly dissected in the present work, the PTEN/VE-cadherin pattern observed here is compatible with the classical view that PTEN loss favors PI3K/AKT pathway activation and promotes a pro-angiogenic, junction-destabilizing endothelial state [27]. In that sense, our data extend an established signaling framework into the setting of breast cancer-derived exosomal communication with endothelial cells. At the same time, because PI3K/AKT activation and other junction-related intermediates were not experimentally measured, this part of the model should be considered biologically plausible rather than formally demonstrated.

The *in vivo* data are consistent with this interpretation. miR-92b-3p overexpression did not produce a strong direct proliferative or migratory effect in cultured breast cancer cells, but it increased xenograft growth, tumor vascularization, and circulating tumor cell counts in mice. The divergence between the *in vitro* tumor cell phenotype and the *in vivo* tumor behavior is informative, because it suggests that miR-92b-3p influences progression mainly by modifying host-tumor interactions, especially the vascular niche. This point strengthens the view that miR-92b-3p is not simply another differentially expressed miRNA, but a candidate mediator of microenvironment-dependent dissemination. Even so, the *in vivo* experiments did not include endothelial-specific genetic manipulation, and the endothelial contribution of the miR-92b-3p/PTEN axis *in vivo* should therefore be regarded as strongly supported, but not formally isolated.

Several limitations should be acknowledged. The clinical cohort was relatively small, derived from a single center, and lacked an independent validation set, so the diagnostic and stage-associated findings should be viewed as preliminary. Some patient characteristics were not fully available, which limited more refined clinicopathological subgroup analyses. The circulating and exosomal miRNA assays were normalized using equal RNA input with U6 as the internal reference; although this approach is widely used, it remains imperfect for liquid samples. The extracellular vesicle preparations were obtained by differential ultracentrifugation and characterized by TEM, NTA, and marker analysis, but this approach yields exosome-enriched vesicles rather than absolutely pure exosomes and cannot fully exclude co-isolated extracellular components. In addition, although the use of both human HMVECs and murine C166 cells supports the reproducibility of the endothelial response across more than one model, cross-species differences may influence miRNA targeting efficiency and junctional regulation, and the C166 data should therefore be interpreted as supportive rather than definitive.

In summary, the present study supports a model in which breast cancer cells release exosome-enriched vesicles carrying miR-92b-3p, which is transferred to vascular endothelial cells and contributes to angiogenesis, endothelial barrier remodeling, and tumor cell dissemination through a PTEN-centered mechanism. The work extends the relevance of miR-92b-3p in breast cancer from a circulating expression signal to a biologically active mediator of tumor-endothelial communication. Further clinical validation and more detailed pathway analysis will be needed to determine how broadly this mechanism operates across breast cancer subtypes and how useful plasma exosomal miR-92b-3p may be in translational settings.

## 5 Conclusions

In conclusion, the present study indicates that miR-92b-3p is enriched in breast cancer-associated plasma and exosome-enriched vesicles and can be transferred from breast cancer cells to vascular endothelial cells through exosome-mediated communication. After uptake by endothelial cells, exosomal miR-92b-3p directly suppresses PTEN and is associated with enhanced angiogenesis, reduced endothelial junctional stability, increased vascular permeability, and facilitated tumor cell dissemination. These findings support a role for the miR-92b-3p/PTEN axis in breast cancer progression and suggest that plasma exosomal miR-92b-3p may serve as a candidate liquid-biopsy marker. Given the limited cohort size and the absence of external validation and deeper downstream pathway analysis, the clinical utility and full mechanistic scope of this axis require further investigation.

## Data Availability

The authors confirm that the data supporting the findings of this study are available within the article and its Supplementary Materials.

## Supplementary Materials

The supplementary material is available online at https://www.techscience.com/doi/10.32604/or.2026.083563/s1.

## Abbreviations

HER2	Human epidermal growth factor receptor 2
TCGA	The cancer genome atlas
PI3K	Phosphatidylinositol 3-kinase
PTEN	Phosphatase and tensin homolog
TEM	Transmission electron microscope
HMVEC	Human microvascular endothelial cells
RT-qPCR	Real-time qPCR
FBS	Fetal bovine serum
PBS	Phosphate-buffered saline
NTA	Nanoparticle tracking analysis
