# Supplementary material for: Breast Cancer Cell-Derived Exosomal miR-92b-3p Promotes Tumor Angiogenesis and Metastasis by Suppressing PTEN in Vascular Endothelial Cells
Source: Oncol Res. 2026 Jul 16;34(8):23. doi: 10.32604/or.2026.083563 (PMC13397346; doi:10.32604/or.2026.083563)
Supplement: Supplementary file 1 [file OncolRes-34-83563-s001.zip › TSP_OR_83563-s001.pdf]

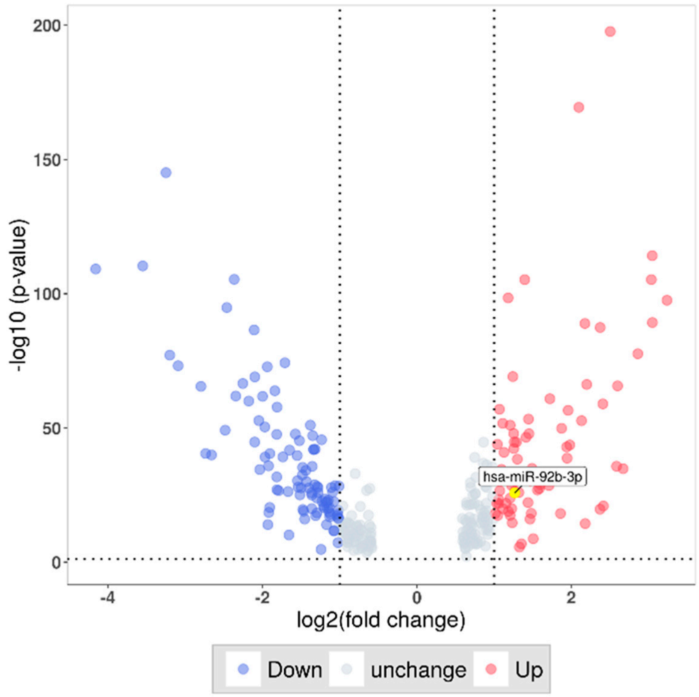

Figure S1. Volcanic map analysis of miRNAs sequencing data of 110 paracancerous tissues and 1200 breast cancer tissue samples in TCGA database. A total of 1260 differentially expressed miRNAs were screened, of which 310 miRNAs were downregulated and 200 miRNAs were upregulated. Compared with adjacent cancer tissues, miR-92b-3p is significantly upregulated in tumor tissues.

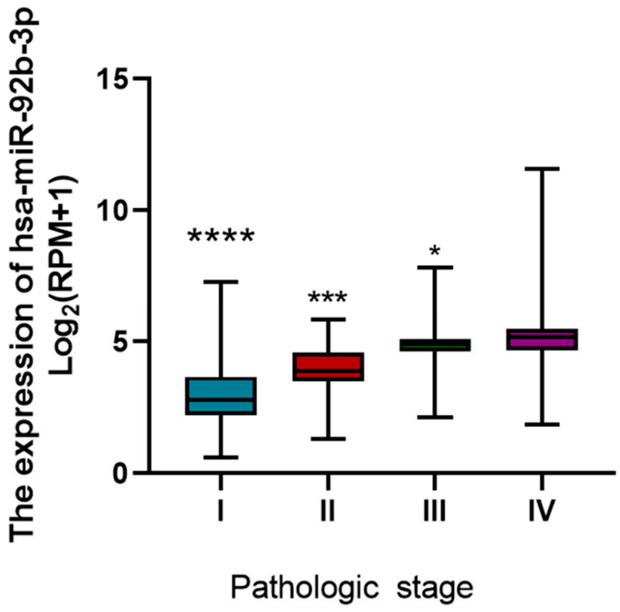

Figure S2. The expression level of miR-92b-3p in stage I-III breast cancer patients (n = 912) was compared with that in stage IV breast cancer patients (n-288) in TCGA database. The difference in * $p < 0.05$, *** $p < 0.001$, **** $p < 0.0001$.

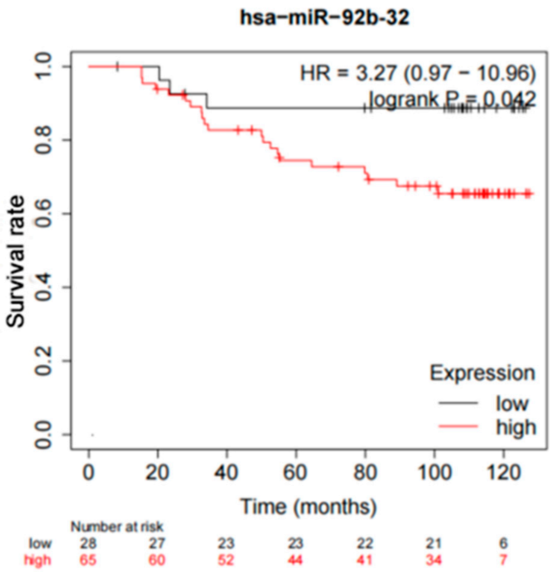

Figure S3. patient survival curves of 110 paracancerous tissues and 1200 breast cancer tissues in TCGA database. According to the median value of mir-92b-3p expression in TCGA database, breast cancer tissues were divided into high expression group and low expression group.
